# Factors influencing the tendency of residual symptoms in patients with depressive disorders: a longitudinal study

**DOI:** 10.1186/s12888-024-05915-9

**Published:** 2024-08-13

**Authors:** Yuwei Li, Dong Wang, Jiexin Fang, Si Zu, Le Xiao, Xuequan Zhu, Gang Wang, Yongdong Hu

**Affiliations:** 1 Department of Clinical Psychology, Beijing Chao-Yang Hospital, Capital Medical University, Beijing, China; 2 The National Clinical Research Center for Mental Disorders & Beijing Key Laboratory of Mental Disorders, Beijing Anding Hospital, Capital Medical University, Beijing, China; 3 Advanced Innovation Center for Human Brain Protection, Capital Medical University, Beijing, China

**Keywords:** Depressive disorder, LCGA, Predictor, Residual symptoms

## Abstract

**Background:**

Residual symptoms of depressive disorders are serious health problems. However, the progression process is hardly predictable due to high heterogeneity of the disease. This study aims to: (1) classify the patterns of changes in residual symptoms based on homogeneous data, and (2) identify potential predictors for these patterns.

**Methods:**

In this study, we conducted a data-driven Latent Class Growth Analysis (LCGA) to identify distinct tendencies of changes in residual symptoms, which were longitudinally quantified using the QIDS-SR16 at baseline and 1/3/6 months post-baseline for depressed patients. The association between baseline characteristics (e.g. clinical features and cognitive functions) and different progression tendencies were also identified.

**Results:**

The tendency of changes in residual symptoms was categorized into four classes: “light residual symptom decline (15.4%)”, “residual symptom disappears (39.3%)”, “steady residual symptom (6.3%)” and “severe residual symptom decline (39.0%)”. We observed that the second class displayed more favorable recuperation outcomes than the rest of patients. The severity, recurrence, polypharmacy, and medication adherence of symptoms are intricately linked to the duration of residual symptoms’ persistence. Additionally, clinical characteristics including sleep disturbances, depressive moods, alterations in appetite or weight, and difficulties with concentration have been identified as significant factors in the recovery process.

**Conclusions:**

Our research findings indicate that certain clinical characteristics in patients with depressive disorders are associated with poor recovery from residual symptoms following acute treatment. This revelation holds significant value in the targeted attention to specific patients and the development of early intervention strategies for residual symptoms accordingly.

## Background

Depressive disorder is a prevalent psychiatric mood disorder, and 17% of people experience an episode of depressive disorder in their lifetime [1]. The depressive disorder has been identified as the second leading cause of disability worldwide, with its chronically relapsing nature presenting significant health concerns across the globe. It is a substantial economic burden, affecting individuals across the lifespan and carrying an overall economic cost of $92.7 billion annually in the United States [2].

To aim of treating depression is to achieve complete remission of symptoms and restore functionality. Nevertheless, even among patients who have achieved full symptomatic remission, depression-related residual symptoms may persist [3, 4], often influenced by multitude of factors [5, 6]. Individuals afflicted with residual symptoms exhibit pronounced psychosocial dysfunction, which subsequently diminishes their quality of life and imposes a dual burden on both the individual and society [7, 8]. The residual symptoms also increase the risk of relapsing to depressive episodes [9]. Targeted management strategies that dovetail with acute treatment can effectively improve residual symptoms and reduce their impact [10]. Mindfulness-based cognitive therapy and targeted pharmacological interventions have been shown to be effective in alleviating residual symptoms [11, 12]. Consequently, the timely recognition of persons who experience more pronounced and durable residual symptoms after their acute treatment can help ensure that interventions are more effective, and lessen the total impact of the condition on its sufferers.

Uniform diagnostic criteria are employed for the diagnosis of depressive disorder. However, there is considerable heterogeneity in the clinical presentation of different patients, and a straightforward diagnosis of presence or absence does not accurately reflect the complexity of the disorder. Previous research utilizing network-based methodologies has delved into the intricate interactions and complex associations among symptoms. The findings indicate that the current classification system’s reliance on aggregating symptoms can result in the loss of critical information, hinting at the heterogeneity of diseases and posing a challenge to the current diagnostic framework [13]. In the study of disease heterogeneity, classifications based on inflammatory markers or genetic factors have revealed the existence of subgroups within populations that fulfill the diagnostic criteria for depression [14, 15]. This more nuanced exploration of depressive disorders, though requiring further validation in terms of accuracy, holds promise in facilitating more targeted interventions for patients, ultimately leading to the efficient utilization of healthcare resources and improved patient outcomes.

In clinical practice, the heterogeneity of diseases is also manifest in the diverse patterns of disease progression. Data-driven methods, such as Latent Class Growth Analysis (LCGA), have the capability to identify distinct tendencies of changes based on repeated dimensional severity data. This technique surpasses earlier solely categorial studies and is more comprehensive in ascertaining progression in residual symptoms. Prior investigations imply that the standard Diagnostic and Statistical Manual of Mental Disorders (DSM) diagnostic tags correlated inadequately (< 50%) to the aggregation of illness trajectories classified by means of a data-driven, bottom-up approach [16]. Thus, utilizing analogous techniques in the demarcation of residual symptoms may result in more sensible inferences in contrast to traditional assessment methods. Previous studies which have employed the conventional classification of residual symptoms have determined that number of illness episodes, insomnia, and time to remission are all prognosticators of the lastingness of residual symptoms [17, 18]. Nonetheless, these conclusions may not be applicable to diverse types of individuals if the variability of clinical courses is not taken into account [19].

This study aims to classify the patterns of changes in residual symptoms of depressed Chinese patients and identify potential predictors for these patterns. Specifically, a data-driven LCGA is used to identify and categorize the trend of changes in residual symptoms and the predictive efficacy of clinical features and cognitive functioning for each pattern is evaluated. Data was collected from multiple medical institutions with participants evaluated over a 6-month follow-up period. The results of this study contribute to the knowledge of residual symptoms in Chinese patients with depressive disorder, and provide a reference point for early identification of treatment options to manage residual symptoms.

## Methods

### Sample

Data for the current study came from a large-scale survey conducted in thirteen cities in China. The study took place between June 2016 to December 2016. All participants enrolled in this study received treatment respectively from sixteen hospitals. This study was approved by the Ethics Committee of Beijing Chaoyang Hospital and Beijing Anding Hospital ((2016) Scientific research No. (37) − 201,640 fs-2) and in line with the principles of the Declaration of Helsinki. All eligible patients provided written informed consent.

#### Inclusion criteria

(1) An age of 18 years or above; (2) Meets the International Classification of Diseases, 10th Edition (ICD-10) diagnosis of a depressive episode (F32) or recurrent depressive disorder (F33); (3) According to the Visual Analogue Scale (VAS), the patient’s current state (depression) is more than 50% recovered from the beginning of the episode; (4) According to the discretion of doctors, antidepressants were the main treatment for patients; (5) After the depressive episode, the patient received antidepressants for 8 weeks (inclusive) to 12 weeks (inclusive), and the cumulative days of withdrawal during the period was less than 14 days; (6) Sufficient level of education and understanding to complete the scale assessment independently with accuracy and speed; (7) At the doctor’s discretion, the patient was able to complete a six-month follow-up.

#### Exclusion criteria

(1) A clear history of previous episodes of mania or hypomania or a diagnosis of bipolar disorder, schizophrenia, schizoaffective disorder, or other disorders accompanying mental disorders; (2) Enrolled in the pre-trial study; (3) Based on the investigator’s judgment, the patient could not follow the study protocol.

### Measures

#### Demographics and clinical features

In this paper, a self-administered demographic and clinical feature scale was employed to obtain data on patients’ sociological characteristics, including gender, age, and education, as well as disease characteristics such as the age of onset and recurrence. These factors were taken into account, as they have the potential to affect the change in residual symptoms in patients or intervene with the results.

#### Brief 16-Item Quick Inventory of Depressive Symptomatology Self-Report (QIDS-SR16)

The QIDS-SR16 questionnaire assesses the presence and strength of significant depression through a 16-item survey [20]. This scale adopts a four-level rating system, considering the severity of depressive symptoms during the past 7 days. The total score ranges from 0 to 27 and is calculated as the sum of the highest score among 1–4 items, the highest score among 6–9 items, the highest score among 15–16 items, and the scores of the remaining items. An elevated score on the scale reflects the greater intensity of depression-related symptoms [21].

#### Patient Health Questionnaire-15 (PHQ-15)

The PHQ-15 is a self-rating scale developed to detect physical symptoms. It could assess the severity of cardiopulmonary, gastrointestinal, pain, and fatigue/general symptoms over the past 4 weeks. The overall score of the PHQ-15 ranges from 0 to 30, and the score of each item ranges from 0 to 3. Kroenke initially validated the PHQ-15 [22], and the Chinese version was tested in Shanghai [23]. Cronbach’s alpha was 0.73, and the test-retest reliability coefficient was 0.75 [24].

#### The 7-item generalized anxiety disorder scale (GAD-7)

The GAD-7 has been validated and widely used in the literature. It could assess the severity of anxiety symptoms [25]. Three-level scoring mode is adopted, and the total score ranges from 0 to 21. Patients with severe anxiety tend to receive higher scores. Psychiatrists and translators apply the Chinese version of the GAD-7 to general hospital outpatients on the Chinese mainland. The results showed that GAD-7 has high sensitivity and specificity [26].

#### Cognitive testing

Digit Symbol Substitution Test (DSST) could assess information processing ability and memory. This test requires subjects to fill in symbols sequentially within the 90s [27]. The Digital Span Test (DST) is a subtest on the Wechsler Intelligence Scale that assesses attention, short-term memory, and working memory [28]. The scoring of it can be divided into three parts: forward, backward, and total scores.

### Research process

The study was carried out in several hospitals at the same time. Participants were assessed at baseline to determine whether they met the criteria. Patients who voluntarily enrolled and completed informed consent were followed up for 6 months. The data, such as demographics, clinical features, medication information, QIDS-SR16, PHQ-15, GAD-7, and cognitive testing, were collected during the baseline period. A follow-up visit was performed at 1,3,6 months, including QIDS-SR16 and medication adherence. Good adherence was defined as “never missed a dose/occasionally missed a dose” and poor adherence as “sometimes missed a dose, missed a dose about half the time, missed a dose frequently, or missed a dose at all” at each visit. During the follow-up phase, participants who exhibited signs of deterioration or refused to participate further were excluded from the study. To guarantee the uniformity of the study, all investigators from each hospital were provided with pre-study training, which included the standard for diagnosis and evaluation. All assessments were conducted by an independent worker who was blinded to this study.

### Statistical analysis

SAS 9.4 and Mplus were used for statistical analysis in this study. LCGA is an algorithm used to identify categories of patients with different disease courses [29]. It starts with one class and could determine the best-fitting model by adding more classes. To determine the optimal model, we have comprehensively evaluated Akaike’s Information Criterion (AIC), Bayesian information criterion (BIC), the Lo–Mendell–Rubin (LMR), entropy, and interpretability of the classes. The lower AIC and BIC value indicate the modal that best described the data [30]. The LMR test provides a *p*-value that indicates whether the k-1 class model is rejected in favor of the k-class model. Entropy, as a measure of classification quality, is the reference of evaluation classification.

The patients were assigned to the best-fitting classes based on relevant outcomes. The one-sample Kolmogorov-Smirnov test was used to test the normality of the measures, and those that conformed to a normal distribution were described using the mean ± standard deviation. Those that did not conform to a normal distribution were expressed using the median [M(Q25, Q75)]. Count data were described using the number of cases (%), and non-missing data were used for the denominator. A two-sided test with 0.05 as the test level was used. We compared the characteristics of the different classifications through two-tailed x2 statistics for categorical variables and one-way analysis of variance (ANOVA) statistics for continuous variables. We also conducted a related analysis. Multivariate, multinomial logistic regression analyses were conducted to examine determinants of class membership, including demographics, clinical features, medication information, and the scores of QIDS-SR16, PHQ-15, GAD-7, DSST and DST. *P* < 0.05 was considered statistically significant.

## Results

### Latent class growth analyses

We excluded 6 participants due to diagnostic modifications and yielded a final analytical sample of 428 participants. Based on the follow-up total QIDS-SR16 scores in the period, the patients were classed for the course of residual symptoms using LCGA. The associated AIC, BIC, entropy and LMR likelihood ratio tests, as well as the percentage of each class of each LCGA model, are itemized in Table 1. The four-class model was selected for further consideration depending on the statistical results, class size, and interpretability of the classes.

**Fig. 1:**
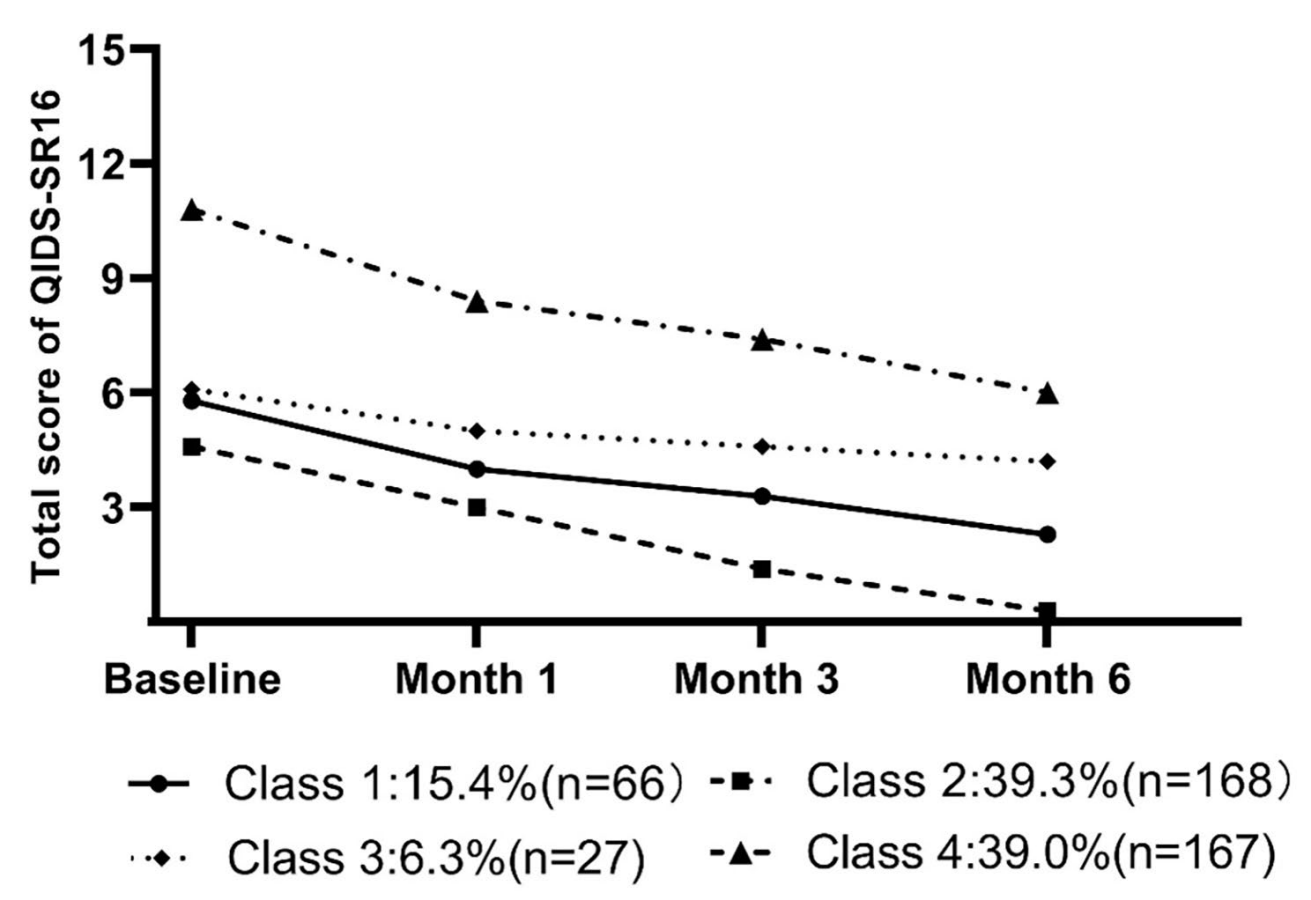
The four-classes model based on QIDS-SR16 in the 6 months following the acute treatment was used to establish trajectories for each of the defined classes. Class 1 = slight residual symptom decline (15.4%); class 2 = residual symptoms disappear (39.3%); class 3 = steady residual symptoms (6.3%); class 4 = severe residual symptom decline (39.0%)

Assessment of four different classes showed different patterns of residual symptoms in patients after acute treatment. As shown in the Fig. 1, class 1 (*n* = 66, 15.4%) has a decline of residual symptoms, and the patients have relatively mild symptoms at baseline. Class 2 (*n* = 168, 39.3%) is characterized by the residual symptoms almost disappearing at the end of follow-up. Class 3 (*n* = 27, 6.3%) continues at a similar level throughout the 6 months. Class 4 (*n* = 167, 39.0%) shows a decline of residual symptoms, but initial depression severity is higher than others.

**Table 1:** Latent Class Growth Analysis on the 6 months residual symptoms scores after acute treatment (*n* = 428)

Class	Maximum likelihood	AIC	BIC	Entropy	LMR test		The proportion of individuals in class				
					2LL	*P*	1	2	3	4	5
Linear											
1											
2	-3550.63	7139.25	7216.38	0.696	416.64	< 0.001	39.0	61.0			
3	-3524.26	7092.45	7181.75	0.748	52.80	0.003	36.0	15.9	50.1		
4	-3500.54	7051.08	7152.56	0.759	47.37	< 0.001	17.3	34.9	7.4	40.5	
5	-3493.26	7042.52	7156.18	0.762	14.56	0.191	6.7	12.7	5.8	34.0	40.9
Quadric											
1											
2	-3551.42	7154.85	7260.38	0.695	416.74	< 0.001	42.1	57.9			
3	-3523.47	7106.93	7228.70	0.751	55.92	0.004	34.4	16.0	49.7		
4	-3498.94	7065.89	7203.90	0.758	49.04	0.017	35.0	7.8	17.2	40.1	
5	-3491.95	7059.90	7214.14	0.784	46.91	0.121	35.0	1.5	16.8	8.0	38.7

AIC, Akaike Information Criterion; BIC, Bayesian Information Criterion; LMR, the Lo–Mendell–Rubin

### Differences between classes

A total of 428 people were counted in four classes based on LCGA results. In the demographic information, there were no significant differences among classes in gender, education years, visit selection, marital status, and residence status (*P* ≥ 0.05). However, there are differences in terms of age, career, and income(*P*<0.05). Compared to individuals in other classes, those in class 3 were more likely to be older, retire, and earn 1000–5000(Table 2).

Many clinical features were also counted and compared. No significant differences were found when comparing classes for the duration of the current episode, treatment duration, prescription of antidepressant medications, use of benzodiazepines in combination, and family history (*P* ≥ 0.05). As for medical history, age of the first episode, physical disorders, and adherence, significant differences were found among the four classes. In class 2, 29.8% had recurrence compared to more than 40% in other classes. The average age of the first episode of class 4 was the smallest among the four classes, only 38.8 years old. As for adherence, class 3 showed better adherence to medication, while class 4 was relatively poor (Table 3).

**Table 2:** Comparison of baseline demographic characteristics between four classes

	Class 1	Class 2	Class 3	Class 4	$F/{\chi }^{2}$	*P*
Gender, n(%)					3.18	0.364
Male	18(27.3)	48(28.6)	9(33.3)	61(36.5)		
Female	48(72.7)	120(71.4)	18(66.7)	106(63.5)		
Age	45.4(12.6)	45.3(14.8)	51.1(13.7)	42.3(14.1)	3.54	0.015
Education years	11.6(4.0)	11.7(4.3)	10.9(3.6)	12.6(4.2)	2.07	0.103
Visit selection					1.60	0.659
General hospitals	39(59.1)	99(58.9)	19(70.4)	96(57.5)		
Specialist hospitals	27(40.9)	69(41.1)	8(29.6)	71(42.5)		
Marital status					12.53	0.185
Married/cohabiting	54(81.8)	131(78.0)	23(85.2)	109(66.1)		
Divorced/separated	3(4.6)	4(2.4)	0(0.0)	8(4.9)		
Widowhood	1(1.5)	6(3.6)	9(5.5)	9(5.5)		
Single	8(12.1)	27(16.1)	39(23.6)	39(23.6)		
Residence status					13.17	0.155
Live alone	3(4.6)	10(6.0)	0(0.0)	20(12.1)		
With family	58(87.9)	146(86.9)	26(96.3)	138(83.6)		
With friend	2(3.0)	7(4.2)	1(3.7)	6(3.6)		
Others	3(4.6)	5(3.0)	0(0.0)	1(0.6)		
Career					28.18	0.021
Student	4(6.1)	13(7.7)	0(0.0)	12(7.3)		
Employment	33(50.0)	85(50.6)	12(44.4)	86(52.1)		
Unemployed	4(6.1)	8(4.8)	0(0.0)	12(7.3)		
Retirement	9(13.6)	36(21.4)	14(51.9)	26(15.8)		
Full-time housework	11(16.7)	21(12.5)	1(3.7)	20(12.1)		
Others	5(7.6)	5(3.0)	0(0.0)	9(5.5)		
Income					18.97	0.025
≤ 1000	16(24.2)	38(22.6)	1(3.7)	44(26.7)		
1000 ~ 5000	34(51.5)	90(53.6)	22(81.5)	80(48.5)		
5000 ~ 10,000	15(22.7)	27(16.1)	2(7.4)	23(13.9)		
≥ 10,000	1(1.5)	13(7.7)	2(7.4)	18(10.9)		

*P*-value from Chi-square test (χ²) and One-way ANOVA

**Table 3:** Comparison of baseline clinical features between four classes

	Class 1	Class 2	Class 3	Class 4	$F/{\chi }^{2}$	*P*
Duration of the current episode (weeks)	21.2(19.4)	22.5(17.6)	18.8(7.2)	20.1(13.7)	0.87	0.459
Medical history					9.97	0.019
Recurrence	30(45.5)	50(29.8)	12(44.4)	75(44.9)		
First	36(54.5)	118(70.2)	15(55.6)	92(55.1)		
Age of first episode	42.9(13.2)	43.3(14.5)	48.6(13.1)	38.8(14.6)	5.32	0.001
Duration of treatment (weeks)	8.5(8,10)	9(8,11)	8.9(8,11.4)	8.9(8,12)	4.38	0.223
Physical disorders	16(24.2)	45(26.8)	14(51.9)	50(29.9)	8.00	0.046
Antidepressant medication					1.19	0.756
Monotherapy	48(72.7)	117(69.6)	19(70.4)	125(74.9)		
Polypharmacy	18(27.3)	51(30.4)	8(29.6)	42(25.2)		
Benzodiazepines	37(56.1)	90(53.6)	18(66.7)	91(54.5)	1.67	0.644
Family history	6(10.3)	18(12.1)	2(9.1)	18(13.0)	0.47	0.925
Adherence	42(63.6)	78(46.4)	22(81.5)	60(35.9)	28.44	< 0.001

*P*-value from Chi-square test (χ²) and One-way ANOVA

The multi-scale approach utilized in this study, including the QIDS-SR16, PHQ-15, GAD-7, DSST, and DST, was employed to measure the symptomology of patients. Further analysis of the QIDS-SR16 was conducted on an item-by-item basis in order to compare results between subjects. Class 4 subjects scored higher in all assessments, excluding those for cognitive functioning (Table 4).

**Table 4:** Comparison of baseline scores between four classes

	Class 1	Class 2	Class 3	Class 4	$F/{\chi }^{2}$	*P*
Total QIDS	5.9(3.1)	4.6(2.7)	6.1(2.2)	10.8(4.6)	92.6	< 0.001
QIDS-1-4	1.4(0.9)	1.2(0.9)	1.5(0.9)	2.1(0.9)	28.4	< 0.001
QIDS-5	0.6(0.7)	0.4(0.6)	0.5(0.6)	1.2(1.0)	28.1	< 0.001
QIDS-6-9	0.8(0.8)	0.6(0.9)	1.1(0.9)	1.4(1.1)	19.8	< 0.001
QIDS-10	0.8(0.8)	0.5(0.6)	0.6(0.6)	1.2(0.8)	34.1	< 0.001
QIDS-11	0.4(0.6)	0.3(0.6)	0.3(0.5)	1(1.0)	26.9	< 0.001
QIDS-12	0.1(0.3)	0(0.2)	0.1(0.3)	0.5(0.8)	19.6	< 0.001
QIDS-13	0.5(0.6)	0.5(0.6)	0.7(0.7)	1.2(1.0)	28.0	< 0.001
QIDS-14	0.6(0.6)	0.6(0.5)	0.7(0.5)	1.1(0.8)	18.0	< 0.001
QIDS-15-16	0.7(0.8)	0.5(0.7)	0.6(0.6)	1.2(0.8)	25.9	< 0.001
PHQ-15	4.7(3.3)	4.4(3.3)	5.1(3.3)	8.5(4.8)	33.5	< 0.001
GAD-7	2.6(2.3)	2.4(2.8)	2.6(2.5)	6.5(4.8)	40.1	< 0.001
DSST	40.8(17.5)	40.4(17.4)	37.1(18.6)	43.5(17.0)	1.55	0.200
DST-Forward	7.6(1.4)	7.5(1.8)	7.1(1.6)	7.9(1.8)	2.33	0.074
DST-Backward	4.8(1.3)	5(1.6)	4.3(1.5)	5.1(1.7)	2.21	0.087

*P*-value from Chi-square test (χ²) and One-way ANOVA

Among the participants, 309 (72.2%) received a single type of antidepressant, while 119 (27.8%) received two types. Within the monotherapy cohort, selective serotonin reuptake inhibitors (SSRIs) constituted the foremost prescribed antidepressants, encompassing over 60% of prescriptions across classes 1 through 4. Escitalopram Oxalate emerged as the most frequently administered SSRIs, while the utilization of serotonin-norepinephrine reuptake inhibitors (SNRIs) surpassed 20% across all classes. Furthermore, a select few patients received exclusive treatment with norepinephrine and specific serotonergic antidepressants (NaSSA). As for polypharmacy, the most frequently administered combinations were SSRIs + SSRIs and SNRIs + NaSSA (Table 5).

**Table 5:** Type of antidepressants that the patients received

Type of antidepressants	Class1	Class2	Class3	Class4
Monotherapy	48	117	19	125
SSRIs(%)	30(62.5)	78(66.7)	14(73.7)	78(62.4)
Escitalopram Oxalate(%)	14(29.2)	42(35.9)	8(42.1)	24(19.2)
Sertraline(%)	7(14.6)	9(7.7)	0(0.0)	22(17.6)
Paroxetine(%)	4(8.3)	10(8.6)	2(10.5)	16(12.8)
Fluoxetine(%)	3(6.3)	4(3.4)	0(0.0)	9(7.2)
Citalopram Hydrobromide(%)	2(4.2)	12(10.3)	3(15.8)	4(3.2)
Fluvoxamine(%)	0(0.0)	1(0.9)	1(5.3)	3(2.4)
SNRIs(%)	15(31.3)	31(26.5)	4(21.1)	44(35.2)
Venlafaxine(%)	8(16.7)	10(8.6)	4(21.1)	30(24.0)
Duloxetine(%)	7(14.6)	21(18.0)	0(0.0)	14(11.2)
NaSSA(%)	2(4.2)	7(6.0)	0(0.0)	2(1.6)
Polypharmacy	18	51	8	42
SSRIs + SSRIs(%)	6(35.3)	18(42.9)	3(42.9)	10(30.3)
SSRIs + SNRIs(%)	1(5.9)	1(2.4)	3(42.9)	4(12.1)
SSRIs + NaSSA(%)	4(23.5)	8(19.1)	0(0.0)	8(24.2)
SNRIs + NaSSA(%)	6(35.3)	13(31.0)	1(14.3)	11(33.3)
SNRIs + SNRIs(%)	0(0.0)	2(4.8)	0(0.0)	0(0.0)

SSRIs: Selective Serotonin Reuptake Inhibitors; SNRIs: Serotonin and Noradrenalin Reuptake Inhibitors; NaSSA: Norepinephrine and Specific Serotonergic Antidepressants

### The relationship between adherence and symptom severity

An examination of the relationship between adherence and symptom severity across the general population and specific classes revealed a positive correlation between the two factors in QIDS-SR16, PHQ-15, and GAD-7 scores among all patients. A detailed analysis of the four classes revealed contrasting findings. In class 1, there was no correlation between severity and adherence. In contrast, class 2 exhibited a significant positive correlation between PHQ-15 and GAD-7 scores with adherence, particularly pronounced at month 6. In class 3, a negative correlation was observed between PHQ-15 and adherence only at month 3. Finally, in class 4, adherence positively correlated with symptom scores in QIDS-SR16 and PHQ-15 at month 3, as well as in GAD-7 at month 6 (Table 6).

**Table 6:** Type of antidepressants that the patients received

	QIDS-SR16	PHQ-15	GAD-7
Total			
Month 1	0.01	0.13*	-0.08
Month 3	0.11*	0.14*	0.06
Month 6	0.17	0.17*	0.19*
Class1			
Month 1	-0.06	0.10	0.03
Month 3	0.02	-0.05	-0.22
Month 6	-0.22	0.04	-0.01
Class2			
Month 1	0.00	0.20*	-0.06
Month 3	0.13	0.12	-0.05
Month 6	0.08	0.20*	0.31*
Class3			
Month 1	0.00	0.04	0.05
Month 3	-0.02	-0.39*	-0.06
Month 6	0.04	-0.22	-0.30
Class4			
Month 1	-0.17	0.12	-0.13
Month 3	0.18*	0.25*	0.17
Month 6	0.18	0.19	0.20*

*P*-value from Correlation Test; *P<0.05

### Baseline predictors of class membership

A multinomial multivariate logistic regression analysis (with reference class 2, indicating residual symptom disappearance) was conducted to assess the effect of baseline characteristics on the residual symptoms persisting after acute treatment. The results of this analysis are shown in Table 7. The result showed that recurrence, adherence and concentration (QIDS-10) was associated with class 1(OR 5.54, 95%CI 1.17–26.12; OR 2.21, 95%CI 1.02–4.76; OR 2.23, 95%CI 1.17–4.24). Recurrence and good adherence were also associated with class 3 (OR 8.85, 95%CI 1.04–75.33; OR 4.59, 95%CI 1.12–18.77). As the change in appetite or weight (QIDS-6-9) increased, the odds for class 3 also increased (OR 3.05,95%CI 1.59–5.82). As for class 4, the income, antidepressant combination, sleep disturbance (QIDS-1-4), depressed mood (QIDS-5), change in appetite or weight (QIDS-6-9), decreased concentration (QIDS-10), and DST-Forward were associated with it (OR 1.75, 95%CI 1.00-3.06; OR 0.29, 95%CI 0.11–0.72; OR 1.87, 95%CI 1.24–2.84; OR 2.22, 95%CI 1.28–3.86; OR 3.01, 95%CI 1.94–4.66; OR 2.83, 95%CI 1.50–5.36; OR 1.48, 95%CI 1.08–2.02).

**Table 7:** Predictors of tendencies in residual symptoms after acute treatment

	Class 1		Class 3		Class 4	
	OR (95% CI)	*P*	OR (95% CI)	*P*	OR (95% CI)	*P*
Duration of current episode	1.01(0.99–1.03)	0.279	1.01(0.97–1.04)	0.772	0.99(0.96–1.01)	0.329
Recurrence	5.54(1.17–26.12)	0.031	8.85(1.04–75.33)	0.046	1.27(0.31–5.23)	0.740
Age of first episode	1.06(0.95–1.17)	0.313	1.05(0.91–1.21)	0.492	0.92(0.83–1.01)	0.092
Duration of treatment	0.79(0.61–1.02)	0.072	1.23(0.82–1.83)	0.317	1.13(0.88–1.46)	0.340
Physical disorders	1.08(0.43–2.68)	0.872	0.27(0.07–1.06)	0.060	0.65(0.25–1.66)	0.368
Polypharmacy	0.91(0.42-2.00)	0.818	0.84(0.25–2.78)	0.777	0.29(0.11–0.72)	0.008
Benzodiazepines	1.02(0.48–2.17)	0.954	0.39(0.11–1.37)	0.144	0.64(0.29–1.40)	0.266
Family history	1.46(0.48–4.49)	0.505	1.26(0.20–8.04)	0.805	1.29(0.42–3.98)	0.663
Adherence	2.21(1.02–4.76)	0.043	4.59(1.12–18.77)	0.034	0.75(0.34–1.64)	0.469
QIDS						
QIDS-1-4	1.07(0.72–1.61)	0.735	1.89(0.97–3.68)	0.062	1.87(1.24–2.84)	0.003
QIDS-5	1.74(0.98–3.09)	0.058	0.86(0.32–2.29)	0.761	2.22(1.28–3.86)	0.005
QIDS-6-9	1.42(0.92–2.18)	0.109	3.05(1.59–5.82)	0.001	3.01(1.94–4.66)	< 0.001
QIDS-10	2.23(1.17–4.24)	0.015	1.65(0.61–4.46)	0.319	2.83(1.50–5.36)	0.001
QIDS-11	1.15(0.61–2.18)	0.663	1.09(0.39–3.03)	0.865	1.71(0.95–3.08)	0.076
QIDS-12	1.56(0.41–6.01)	0.517	0.61(0.07–5.48)	0.661	1.26(0.40–3.98)	0.698
QIDS-13	0.66(0.34–1.30)	0.232	2.03(0.81–5.09)	0.130	1.45(0.82–2.57)	0.199
QIDS-14	0.54(0.26–1.14)	0.106	1.37(0.49–3.88)	0.548	1.12(0.57–2.19)	0.747
QIDS-15-16	1.72(0.99-3.00)	0.055	0.67(0.25–1.74)	0.407	1.09(0.63–1.86)	0.763
PHQ-15	1.02(0.90–1.16)	0.767	1.06(0.87–1.30)	0.537	1.13(1.00-1.27)	0.057
GAD-7	0.88(0.74–1.04)	0.126	0.95(0.74–1.22)	0.697	1.06(0.91–1.23)	0.452
DSST	1.00(0.97–1.03)	0.998	1.02(0.97–1.07)	0.517	1.02(0.98–1.05)	0.294
DST-Forward	1.19(0.89–1.59)	0.245	0.92(0.57–1.49)	0.730	1.48(1.08–2.02)	0.015
DST-Backward	1.01(0.73–1.40)	0.942	0.69(0.41–1.16)	0.166	0.85(0.63–1.16)	0.302

*P*-value from a multivariate logistic regression analysis, excluding the effect of general demographic information

## Discussion

Depression is one of the most prevalent mental disorders and its effects can be recurrent or long-lasting. The residual symptoms after acute treatment would further increase the risk of recurrence, as demonstrated by Verhoeven et al [31]. Furthermore, depression is a leading cause of disability, with a significantly higher rate of disability observed among those with residual symptoms [32]. Therefore, it is clinically significant to determine the trajectory of changes in residual symptoms and to analyze the factors influencing it to provide clues for the targeted selection of patients for early intervention of residual symptoms.

The current study aimed to use LCGA to determine the patterns of changes based on depressive residual symptoms after acute treatment. Four classes were identified, including “slight residual symptom decline”, “residual symptoms disappear”, “steady residual symptoms”, and “severe residual symptom decline”. The patient whose residual symptoms largely disappeared at the end of the follow-up period is considered the best-recovered pattern. Accordingly, it is paramount to identify pertinent prognostic markers to discern among the multiple potential patterns and to allow better outcomes to be attained even in patients that do not fit the best-recovered tendency, owing to early intervention.

A cross-sectional comparison was conducted to identify differences among patients belonging to each class. Data from this study indicate that patients exhibiting the best recovery outcomes had the lowest recurrence rate of depressive disorder. Moreover, patients displaying best-recovered residual symptoms were observed to have a relatively lower degree of symptom severity. Based on the above, residual symptoms may be more rapidly resolved in first-episode patients, particularly those with mild symptomatology. In this study, the second step was to compare the classes with a relatively poor trend in residual symptoms to the “residual symptoms disappear” class to assess the factors that predict rapid remission. The “slight residual symptom decline” class, “steady residual symptoms” class, and “residual symptoms disappear” class had similar lower levels of symptoms at baseline. However, the latter had essentially disappeared at the end of the follow-up. Our comparison of the three classes revealed that recurrent episodes and adherence to prescribed treatments were significantly associated with poorer disease recovery and overall health outcomes. In the “severe residual symptom decline” class, which displayed varying severity levels at baseline, polypharmacy therapy appeared to be a more significant protective factor, although the study design does not permit making definitive causal assertions.

Previous studies have demonstrated that recurrent patients have a more grave presentation of depressive symptoms and diminished quality of life than those with first-episode depression [8]. Results from the present study further suggest that first-episode patients present with a more favorable trend of changes in residual symptom relief. These differences in clinical features may be attributed to differences in biological specificity. Cognitive deficits were widespread among patients, with recurrent depression patients having poorer verbal and visual learning performance than first-episode patients [33]. Sun et al. conducted a study investigating the differences in neural activity between the first episode and recurrent depression [34]. Their results highlighted that those with recurrent depression exhibited more widely distributed and complex neural mechanisms when compared to their counterparts with first-episode depression. This potential alteration in the neural organization may account for the discrepancy in the remission of residual symptoms between individuals with no significant severity distinction.

Adherence may have a close relationship with residual symptoms. Poor adherence with psychotropic medication has been linked with an array of adverse outcomes, including exacerbation of the condition, diminished treatment efficacy, or suboptimal response to subsequent treatment. Further, documented problems resulting from poor adherence included re-hospitalization, impaired quality of life, increased comorbidity, and elevated risk of suicide [35–37]. This study examined the adherence of follow-up participants, and the results were contrary to previous studies [38]. We further investigated the relationship between adherence and the severity of the disease. Our study uncovered a positive correlation between depression and adherence, particularly in the ‘severe residual symptom decline’ class. This indicates that patients with more severe symptoms tend to exhibit improved adherence, probably owing to the symptom relief they experience through medication, leading to a heightened perception of medication effectiveness and fostering better adherence. Regarding somatic symptoms, we observed a positive association with adherence in the ‘residual symptoms disappear’ and ‘severe residual symptom decline’ classes. This is likely attributed to patients’ belief that medication can address their existing somatic symptoms, thus enhancing adherence. However, during the ‘steady residual symptoms’ class, where depressive symptoms persist despite medication, somatic symptoms may hinder patients’ adherence. With regard to anxiety, a positive correlation with adherence was noted in the sixth month of the ‘residual symptoms disappear’ and ‘severe residual symptom decline’ classes. This can be explained by anxious patients’ heightened concern for their wellbeing, motivating them to adhere more rigorously to medical advice.

It is well-established that major depressive disorder can cause more severe effects than mild depression [39]. Severity is an essential consideration in treatment decision-making for depression and has received attention in acute treatment. This study focused on the severity of symptoms in patients after acute treatment. Results indicated that subjects with milder residual symptoms experienced optimal relief by the conclusion of the follow-up period. A recent study comprising 15,661 participants examined initial symptom severity as a risk factor for unfavorable depression trajectories, with findings congruent to those presented in the present study [40]. This phenomenon can be partially explained by the fact that patients with milder residual symptoms may have diminished symptomology at the outset of their treatment and the medications administered may also be more effective. Clinical practice indicates that physicians generally refer to prior treatments and the severity of a given ailment when selecting medications [41]. Moreover, unlike the single-focused mechanism of monotherapy, polypharmacy enables comprehensive, multi-dimensional treatment for patients with severe conditions. Consequently, in the category of “severe residual symptoms and decline,” patients undergoing polypharmacy tend to receive more adequate care, leading to a faster alleviation of residual symptoms.

Clinical features of depressive symptoms were found to be predictive of the trend of changes in residual symptoms, with sleep disturbance, depressed mood, changes in appetite or weight, and decreased concentration identified as risk factors for worse residual symptom trends. Other factors, including outlook, suicidal ideation, involvement, energy/fatigability, and psychomotor, did not significantly affect. In other words, a patient who continues to have sleep problems, depressed mood, appetite or weight changes, and abnormal concentration after acute treatment is more likely to suffer from long-term residual symptoms.

The relationship between insomnia and depression has received attention. A previous study indicated that insomnia could disrupt trends in depressive symptomatology, and treating insomnia in patients with depression was demonstrated to affect mood positively [42]. In addition, chronic sleep deprivation and insomnia have been observed as external stressors and could lead to depression [43]. A dose-response relationship was established between greater improvements in sleep quality and more significant improvements in mental health [44]. The current study further looked at the possible role of insomnia in the change of residual symptoms. Depressive disorder is primarily characterized by depressed mood, and higher scores typically reflect greater severity of the illness. It is thus thought to impede recovery from residual symptoms as much as the disease’s severity level. Weight/appetite changes are frequently observed in depressed individuals [45]. This may be related to fluctuations in ghrelin secretion [46]. This study observed a potential correlation with residual symptom trends; however, the exact contributing factors warrant further investigation. Concentration is an additional symptom factor that is closely linked to residual symptoms. The DST was also used to evaluate individual attention. However, the correlation between these two factors and the alleviation of residual symptoms is inverse; specifically, poorer concentration or a higher DST score tends to perpetuate the persistence of residual symptoms. This finding is attributable to the rough manner of assessing concentration and the potential confounding effects of memory embedded within the DST score. The results additionally suggest that the impact of concentration on changes in residual symptoms of depression might be further explored.

Moreover, the findings of this study did not reveal any noteworthy relationship between somatic symptoms and anxiety in relation to changes in residual symptoms among patients with depressive disorders following acute treatment. Previous research has documented that the severity and duration of somatic symptoms are associated with comorbid depression [47]. This correlation has been attributed to off-kilter serotoninergic and noradrenergic neurotransmission [48]. Anxiety, which often occurs concomitantly with depression, can lead to poorer functional outcomes and augmented resistance to first-line treatments. Unexpectedly, the current study failed to uncover any additional correlations in the evolution of residual symptoms.

This multi-center study aimed to investigate the changing trend of residual symptoms and the predictive factors of different trends in Chinese patients with depression. The presence of residual symptoms of depression can lead to further deterioration of social functioning and timely intervention based on tailored treatments may effectively reduce the burden brought by residual symptoms of depression. Nevertheless, given the limited resources in the therapy, it is likely that early interventions may be more beneficial to those suffering from longer-term residual symptoms. Therefore, the conclusions of this study can be used to inform clinical practice to increase clinicians’ awareness of particular patients and enable more targeted and cost-effective interventions. In clinical practice, for patients exhibiting persistent depressive symptoms, particularly those involving sleep, mood, appetite, and attention issues, following acute-phase treatment, clinicians must be cognizant of the possibility that residual symptoms may persist for over six months. It is imperative to engage patients thoroughly in discussions, ensuring they have a comprehensive understanding of their condition. Additionally, for patients who exhibit significant improvement following acute-phase treatment, particular attention should be paid to those with a history of relapse or persistent dietary and attentional issues. Health education should be administered to equip them with strategies for managing physical discomfort, and adherence to medication schedules should be emphasized.

Notwithstanding its rigorous nature, this investigation is subject to certain limitations. Despite a considerable overall sample size, the stratification of data revealed that a specific group comprised only 27 patients, thereby hampering the generalization of findings pertaining to that subgroup. Moreover, the follow-up protocol was confined to four discrete time points, potentially overlooking critical aspects of the longitudinal progression of residual symptoms. Additionally, the reliance on clinicians’ subjective assessments in determining follow-up adherence criteria may have introduced bias in patient selection. Furthermore, the inherent challenges of observational studies hinder the establishment of definitive causal relationships. Given the relative obsolescence of the data in this study, future endeavors should build upon current clinical realities, exploring further and delving deeper into additional influential factors, such as personality traits, quality of life, and stigma, to achieve a more comprehensive understanding of the evolutionary dynamics of residual symptoms.

## Conclusions

This study aims to classify the patterns of changes in residual symptoms in Chinese patients with depressive disorders and to identify potential predictors (e.g. clinical features and cognitive functions) for these patterns. Results suggest that there are four trends of changes in residual symptoms after acute treatment, including “slight residual symptom decline”, “residual symptoms disappear”, “steady residual symptoms” and “severe residual symptom decline”. The severity, recurrence, polypharmacy, and medication adherence of symptoms are closely correlated with the duration of residual symptoms’ persistence. Additionally, clinical characteristics including sleep disturbances, depressive moods, alterations in appetite or weight, and difficulties with concentration have been identified as significant factors in the recovery process. Given the identified predictors, more aggressive intervention strategies should be implemented for those in order to minimize the potential damage caused by residual symptoms. In summary, this study highlighted the importance of considering the unique trends of changes in residual symptoms during clinical practice and suggested the provision of early intervention, comprising psychotherapy, for patients with particular characteristics to promote the dissipation of residual symptoms.

## Data Availability

The data in this study is not publicly available due to ethical approval and confidentiality agreements made with participants but are available from the corresponding author upon reasonable request.

## Abbreviations

AIC: Akaike’s Information Criterion
ANOVA: Analysis of variance
BIC: Bayesian information criterion
DSM: Diagnostic and Statistical Manual of Mental Disorders
DSST: Digit Symbol Substitution Test
DST: Digital Span Test
GAD-7: 7-item Generalized Anxiety Disorder scale
ICD-10: International Classification of Diseases, 10th Edition
LCGA: Latent Class Growth Analysis
LMR: Lo–Mendell–Rubin Likelihood Ratio Test
NaSSA: Norepinephrine and Specific Serotonergic Antidepressants
PHQ-15: Patient Health Questionnaire-15
QIDS-SR16: Brief 16-Item Quick Inventory of Depressive Symptomatology Self-Report
SSRIs: Selective Serotonin Reuptake Inhibitors
SNRIs: Serotonin and Noradrenalin Reuptake Inhibitors
VAS: Visual Analogue Scale
